# Prevalence of fimA genotypes of Porphyromonas gingivalis and other periodontal bacteria in a Spanish population with chronic periodontitis

**DOI:** 10.4317/medoral.17009

**Published:** 2012-05-01

**Authors:** Miriam Puig-Silla, Francisco Dasí-Fernánde, José-María Montiel-Company, José-Manuel Almerich-Silla

**Affiliations:** 1 BDS. Department of Stomatology, University of Valencia. Spain; 2 PhD. Fundación Investigación Hospital Clínico Universitario de Valencia/INCLIVA. Valencia. Spain; 3 PhD Dentistry. Teaching Assistant. Department of Stomatology, University of Valencia. Spain; 4 PhD Dentistry. Tenured Lecturer. Department of Stomatology, University of Valencia. Spain

## Abstract

Objectives: The aim of this study was to determine the prevalence of the different fimA genotypes of Porphyromonas gingivalis in adult Spanish patients with chronic periodontitis, patients with gingivitis and periodontally healthy subjects, and the relationship between these genotypes and other periodontopathogenic bacteria. Study design: Samples of subgingival plaque were taken from 86 patients (33 with chronic periodontitis, 16 with gingivitis, and 37 periodontally healthy) in the course of a full periodontal examination. PCR was employed to determine the presence of the 6 fimA genotypes of Porphyromonas gingivalis (I-V and Ib) and of Aggregatibacter actinomycetemcomitans, Tannerella forsythia and Treponema denticola. Results: Porphyromonas gingivalis fimA genotypes II and Ib were present in significantly higher percentages in periodontal patients (39.4% and 12.1% respectively) than in healthy or gingivitis subjects. The prevalence of Tannerella forsythia, Treponema denticola, and Porphyromonas gingivalis fimA genotype IV was significantly higher in the group that presented bleeding greater than 30%. A positive correlation was found between Porphyromonas gingivalis fimA genotype IV and Treponema denticola. Conclusions: A strong association between Porphyromonas gingivalis fimA genotypes II and Ib and chronic periodontitis exists in the Spanish population. The most prevalent genotype in periodontal patients is II.

** Key words:**Periodontitis, Porphyromonas gingivalis, fimA genotype, periodontal bacteria, polymerase chain reaction.

## Introduction

Chronic periodontitis is a disease that affects the periodontal tissues and leads to the loss of alveolar bone (1). The composition of subgingival plaque is complex and has been the subject of numerous studies, as the presence of certain bacteria is associated with worse periodontal status, greater pocket depth and higher bleeding indices (2). *Porphyromonas gingivalis* is considered one of the main agents causing different types of periodontal disease, including chronic periodontitis (3). The virulence of *Porphyromonas gingivalis*, a gram-negative anaerobic bacterium, is attributed to its various surface components, such as fimbriae, lipopolysaccharides and proteases. This surface makes it possible for the bacterium to interact with the external medium and facilitates its growth, nutrient acquisition, colonization, and formation of a biofilm that protects it against the host’s defences (4,5).

Amano et al. (3,6) concluded that *P. gingivalis* can be classified into five genotypes according to genomic differences in the *fimA* gene which codes fimbrillin, a protein of the major fimbriae. Subsequently, Nakagawa et al. (7) discovered a new variant of the *fimA* gene, which they named Ib because it bore a great resemblance to genotype I.

P. gingivalis is frequently found in patients with periodontal disease but has also been observed, although to a lesser extent, in periodontally healthy patients (1). In recent years, studies have been conducted to evaluate the relationship between the different *P. gingivalis* genotypes and periodontal pathogenesis. Genotype II has been observed to be more prevalent in periodontal patients and to be associated with more aggressive forms of the disease (8). Some authors attribute this relationship to its possession of greater adhesiveness and invasiveness (8), while others consider that a high capacity for colonization and ability to evade the host’s defences are responsible (9). The prevalence of the different genotypes has been studied in different populations: Caucasian, Brazilian, Japanese, Chinese and Mexican (1,10-13).

The aim of this study was to determine the prevalence of the different *fimA* genotypes of *P. gingivalis* in adult patients with chronic periodontitis and periodontally healthy subjects, and their relationship with certain clinical signs of this disease such as gingival bleeding. The association of these genotypes with the presence of other periodontopathogenic bacteria such as *Aggrega-tibacter actinomycetemcomitans, Tannerella forsythia* and *Treponema denticola* was also investigated.

## Material and Methods

-Study population

86 patients aged between 25 and 50 years attending the University of Valencia Dental Clinic were included in the study. The patients were informed about the study and took part voluntarily after giving their informed consent. The study design and protocol were both approved by the University of Valencia Ethics Committee.

The patients were divided into three groups depending on their periodontal status. The first group was composed of periodontally healthy patients (n=37). Their mean age was 40.68 years, at least 20 teeth were present excluding third molars, and nowhere did they present any pocket depth greater than 3 mm or loss of periodontal attachment greater than 1 mm (1,14).

The second group was made up of 16 patients with a mean age of 38.81 years. They were classed as having gingivitis, as they met the same conditions as the first group but presented a bleeding index in excess of 30% (11). The third group, comprising the patients with chronic periodontitis, numbered 33 patients with a mean age of 43.39 years who presented at least 4 zones with a probing depth of 5 mm or more and loss of periodontal attachment of 2 mm or more (15).

Patients with early periodontitis, pregnant women and those with drug-induced gingival hyperplasia, or who had taken antibiotics in the previous six months, or who were taking anti-inflammatory medication to treat a chronic condition were excluded from the study, as were patients with HIV infection, Type I or II diabetes mellitus, coronary heart disease, rheumatoid conditions, lupus erythmatosus, Behçet’s syndrome, Crohn’s disease, herpetic gingivostomatitis, pemphigous or oral pemphigoid.

-Clinical examinations and sample collection

A full examination of the entire mouth of each patient was conducted, employing a WHO periodontal probe (PCP11.5B, Hu Friedy, Chicago, IL, USA), and the following were recorded at 6 sites on each tooth (15): bacterial plaque, bleeding, pocket depth, and loss of periodontal attachment. The patients were also classified as ‘non-smokers’, ‘ex-smokers’ or ‘current smokers’. Those who had stopped smoking at least 6 months before the study began were considered ‘ex-smokers’. Those who had stopped smoking less than 6 months previously were included in the ‘current smokers’ group.

The subgingival plaque samples were obtained from the deepest pocket at Ramfjord teeth in the periodontal patients and in the mesiolabial area of a Ramfjord molar in the healthy subjects (1). The supragingival plaque was first removed with a sterile Gracey curette, employed with care to avoid bleeding. Three sterile paper points were inserted as deeply as possible into the gingival groove, left for 15 seconds, removed, and placed in a sterile Eppendorf tube. The bacterial DNA was extracted immediately using the Wizard SV DNA Purification System (Promega Cat. # A2360) in accordance with the manufacturer’s instructions. Following extraction, the DNA samples were stored at -20°C until determination took place.

-Polymerase chain reaction (PCR)

P. gingivalis detection was carried out by PCR according to the method described by Zhao et al. (1). To genotype the *fimA* gene, the specific primers for each subtype described by Amano et al. and Nakagawa et al. (3,7) were used.

The PCR reaction was performed with 100 ng of the bacterial DNA extracted in the previous step, 200 µM of each of the dNTPs, 3 mM MgCl2, 50 pmol of each primer and 0.5 U of AmpliTaq Gold (Applied Biosystems®). The PCR conditions were: an initial taq polymerase activation step at 95°C for 10 minutes, followed by 40 denaturing cycles at 94°C for 30 seconds, annealing at 58°C for 30 seconds, elongation at 72°C for 30 seconds, and lastly a single final elongation step at 72°C for 7 minutes. The PCR products obtained in each reaction were cast in 1.5% agarose gels, stained with ethidium bromide, and viewed by electrophoresis.

Samples in which types I and II were both found were amplified further using specific primers for type Ib. The resulting amplicons were digested with RsaI, cast in 1.5% agarose gels stained with ethidium bromide and viewed by electrophoresis. Samples in which 2 fragments appeared were considered type Ib (7). The positive controls employed were DNA extracted from *P. gingivalis* ATCC33277 and *P. gingivalis* W83.

-Statistical analyses

A chi-square test was used to analyze the comparative prevalence of bacteria and *fimA* genotypes between groups, and a linear-by-linear association test to determine the linear tendency. A p-value of <0.05 was considered significant.

Computations were carried out by SPSS 18.0 statistical analysis software (SPSS Inc., Chicago, IL USA).

## Results

The distribution of the sample by mean age, gender or smoking did not differ significantly between the healthy, gingivitis and periodontitis groups ( Table 1).

**Table 1:**
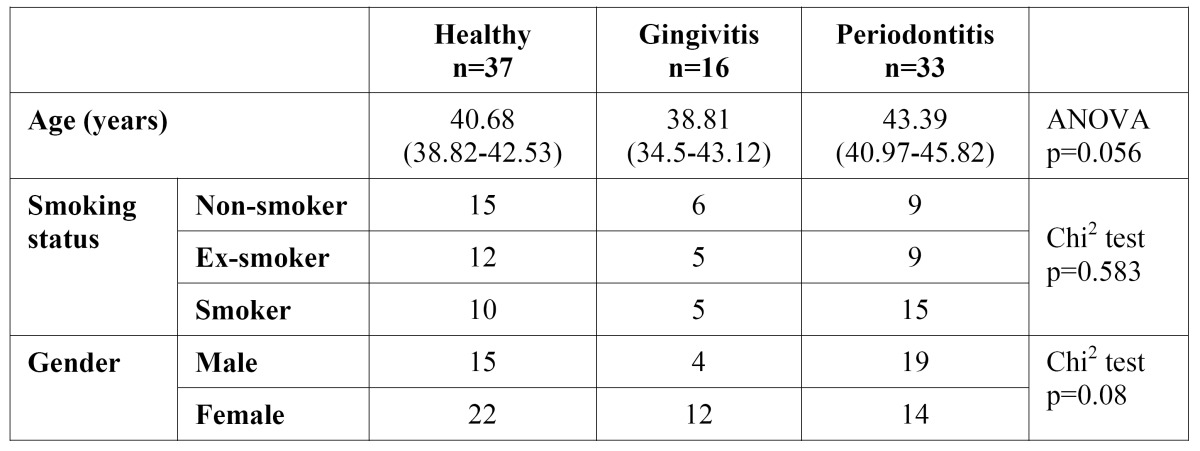
Sample distribution by age, gender and smoking status.

 Table 2 shows the prevalence of the different bacteria found in the subgingival plaque. The three periodontal status groups presented significant differences in rates of *Porphyromonas gingivalis, Aggregatibacter actinomycetemcomitans, Treponema denticola, Tannerella forsythia* and the ‘red complex’, which increased significantly as periodontal condition worsened and reached their highest point in the chronic periodontitis patients. The percentages of *Tannerella forsythia* (69.7%) and *Porphyromonas gingivalis* (66.7%) were higher than those of *Aggregatibacter actinomycetemcomitans* (33.3%) and *Treponema denticola* (48.5%) in the periodontal disease patients. The ‘red complex’ appeared in 30.3% of the sample.

**Table 2:**
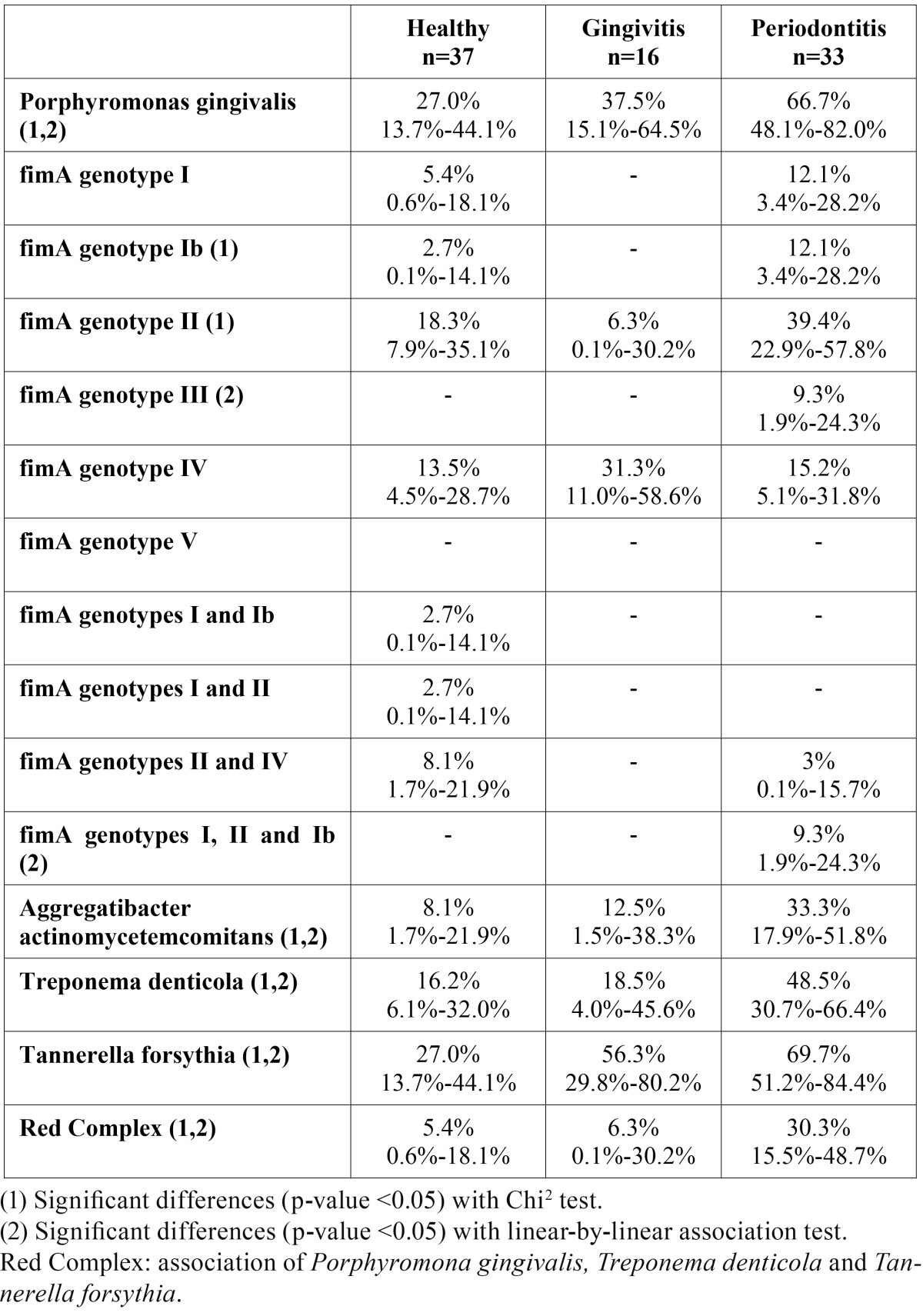
Prevalence of Porphyromonas gingivalis fimA genotypes and periodontal bacteria by periodontal status group.

As regards *Porphyromonas gingivalis fimA* genotypes, genotypes II (39.4%) and Ib (12.1%) were present in significantly higher percentages in the periodontitis patients than in the healthy or gingivitis groups. Genotype IV was more prevalent in the gingivitis group (31.3%), although the differences were not significant. No significant differences in the prevalence of periodontal bacteria or of the different *fimA* genotypes of *Porphyromonas gingivalis* were observed in relation to smoking or gender.

On examining the association between the different *fimA* genotypes of *P. gingivalis* and the other three periodontal bacteria, the only statistically significant positive correlation was between *T. denticola* and *P. gingivalis fimA* genotype IV (Pearson’s coefficient = 0.43), while a statistically significant negative correlation was found between *T. denticola* and *P. gingivalis fimA* genotype Ib (Pearson’s coefficient = 0.36).

The prevalence of *Tannerella forsythia, Treponema denticola* and *Porphyromonas gingivalis fimA* genotype IV was significantly higher in the group that presented bleeding greater than 30%. *Tannerella forsythia, Treponema denticola, Porphyromonas gingivalis* and the ‘red complex’ all showed a positive linear association with bleeding gums ( Table 3).

**Table 3:**
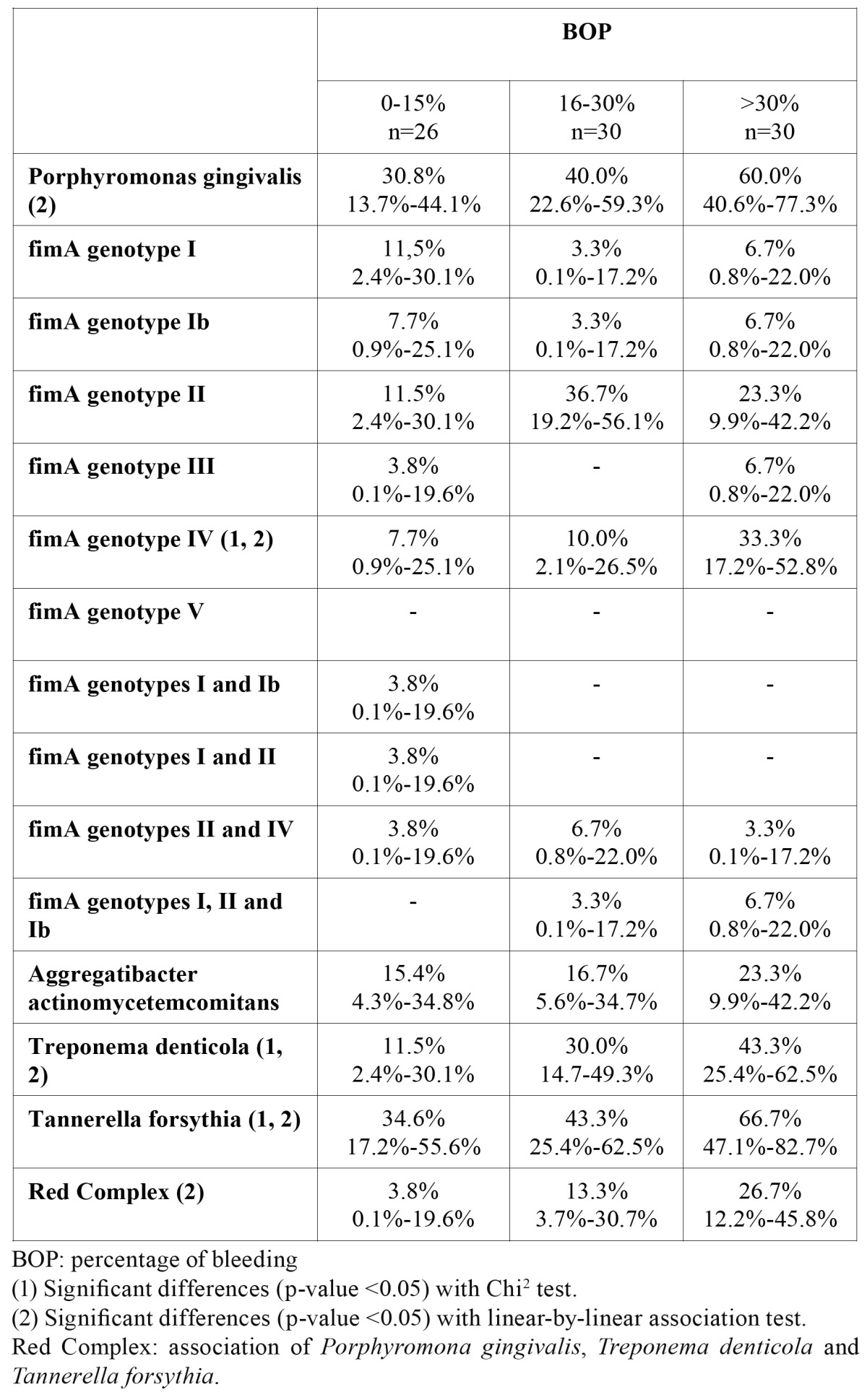
Prevalence of Porphyromonas gingivalis fimA genotypes and periodontal bacteria by percentage of bleeding.

## Discussion

A number of epidemiological studies report that *P. gingivalis* is very frequently present in the subgingival plaque of periodontal patients, ranging from 50.3% to 89.4% of cases (1,3,6,10,11,15). However, it has been demonstrated that this bacterium does not appear exclusively in periodontal patients but is also present in the subgingival plaque of periodontally healthy patients, although to a lesser extent, varying between 22.1% and 36.8% (1,3,16,17). The present study detected P. gingivalis in 66.7% of the patients with chronic periodontitis and 27% of the healthy subjects. Both these percentages fall within the ranges mentioned above.

Many variations in the distribution of *P. gingivalis fimA* genotypes are found, depending on the population being examined, but one fact is repeated in every study: the greater prevalence of *fimA* genotype II in patients with chronic periodontitis. In the German study, the most prevalent genotype in periodontal patients was II, followed by I and IV (10). Misasildis et al. (11) also found that genotype II was the most prevalent in Brazilian periodontal patients, but in this case it was followed by Ib. Similar findings have been reported in studies of Asian patients. In both the Chinese and Japanese studies, genotype II was the most frequent in chronic periodontitis patients, although differences were found in the prevalence of the other genotypes. The present study also determined that genotype II was the most prevalent in the periodontitis patients group, followed by genotype IV. This is a similar result to that of Zhao et al. (1) and differs from that encountered in other populations. However, on comparing the genotype frequencies in periodontitis patients with those of the healthy and gingivitis patients, the statistically significant differences were found in genotypes II and Ib. It should be pointed out that genotype V was not found in any of the patients in the present study. While other authors have detected genotype V, although at very low frequencies, Missaildis et al. (11) did not find it either. Nowadays, these variations are attributed to ethnic and geographical differences (1,11). Indeed, certain studies show marked variations in the subgingival plaque composition of chronic periodontitis patients from different countries who share the same conditions of age, pocket depth, gender and exposure to smoking (15,18,19).

Focusing on genotype II, its frequency in chronic periodontitis patients in this study was 39.4%, far higher than that observed in the healthy subjects. This proportion matches that found in the Brazilian population (39.3%) (11) and is very similar to the fre-quencies observed in other populations such as in Germany (38.2%) (10), China (43.6%) (1) and Japan (46.7%) (12). The greater virulence of genotype II has been studied by several authors, who have found that because of its adhesiveness and invasiveness it is a key determining factor in the virulence of *P. gingivalis* (20).

P. gingivalis, *T. forsythia*, and *T. denticola* are the bacteria that form the classic ‘red complex’ described by Socransky et al. (2). Together with *A. actinomycetem-comitans*, they are the bacteria most frequently found in patients with periodontal disease (21). In vitro studies have also encountered synergies between some of these bacteria (22). Wara-aswapati et al. (23) found a high frequency of the three ‘red complex’ bacteria in the moderate to severe periodontitis patients group, whereas *A. actinomycetemcomitans* was only present in 35% of the patients. In the present study, all four bacteria were present at significantly higher rates in periodontitis patients than in the healthy subjects, particularly *T. forsythia* (69.7%) and *P. gingivalis* (66.7%), although *A. actinomycetemcomitans* was only detected in 33.3% of the chronic periodontitis patients, and a greater presence of the ‘red complex’ was found in patients with chronic periodontitis. Furthermore, the association of the three bacteria that form this complex rose linearly as the periodontal condition worsened. A significant association between the ‘red complex’ and the severity of the disease was also found in the Thai population (23).

In the gingivitis patients group, the present study only found *fimA* genotypes II and IV, the two that were detected most frequently by Zhao et al. (1) in all the patients who presented gingival bleeding on probing. These results differ from those obtained by Fujise et al. (24), who observed that the presence of genotype I was associated with a higher occurrence of bleeding following treatment for periodontitis. On comparing the presence of the bacteria with the percentage of bleeding on probing in all the patients in the present study, a significant association was found between *T. denticola, T forsythia* and *P. gingivalis fimA* genotype IV and this clinical sign.

Smoking modifies the host’s inflammatory and immune responses, affecting his or her defence cells (25,26). The present study found no differences in the distribution of the bacteria studied or of *P. gingivalis fimA* genotypes on comparing smokers with ex-smokers and non-smokers, whether in the periodontitis, gingivitis, or healthy patient group (data not shown). Some previous studies have encountered no differences in the quantities of *P. gingivalis* obtained from subgingival samples from smokers and non-smokers (27). Others, however, have found certain bacteria such as *P. gingivalis* and *T. denticola* to be more prevalent in smokers than in ex-smokers or non-smokers (28).

On analyzing the results of the present study, a strong association was found between the presence of *P. gingivalis fimA* genotypes II and Ib and chronic periodontitis in the Spanish population, with genotype II being the most prevalent in the periodontal patients. This result suggests that further research is needed to investigate their pathogenicity.
